# The effectiveness of a digital shared decision-making tool in hormonal contraception during clinical assessment: study protocol of a randomized controlled trial in Spain

**DOI:** 10.1186/s12889-019-7572-9

**Published:** 2019-09-04

**Authors:** Maria Inmaculada de Molina-Férnandez, Laia Raigal-Aran, Miriam de la Flor-Lopez, Paula Prata, Isabel Font-Jimenez, Francesc Valls-Fonayet, Gemma March-Jardi, Ramon Escuriet-Peiro, Lourdes Rubio-Rico

**Affiliations:** 1 Nursing Department, Campus Catalunya, Universitat Rovira i Virgili, Av/ Catalunya, 35, 43002 Tarragona, Spain; 2 Medicine Department, Universitat Rovira i Virgili, C/ Dr. Mallafrè Guasch, 4, 43005 Tarragona, Spain; 3 Escola Superior de Enfermagem do Porto, Portugal, Rua Bernardino de Almeida, 4200-072 Porto, Portugal; 4 Universitat Rovira i Virgili, Campus Catalunya, Universitat Rovira i Virgili, Av/ Catalunya, 35, 43002 Tarragona, Spain; 5 Institut Català de la Salut, Primary Care Unit. CAP Jaume I, C/ Jaume i, 45-49, 43005 Tarragona, Spain; 6 Àrea d’Atenció Sanitària. Gerència de Salut i Atenció Integrada, Servei Català de la Salut | Generalitat de Catalunya, Travessera de les Corts, 131-159 | Pavelló Ave Maria, |08028 Barcelona, Spain

**Keywords:** Study protocol, Clinical trial, Contraception, Treatment adherence, E-health, Shared decision-making tool

## Abstract

**Background:**

Decision-making tools represent a paradigm shift in the relationship between the clinician and the user/patient. Some of their advantages include patient commitment, the promotion of preferences and values, and increased treatment adherence.

This study protocol aims to assess the effectiveness of a decision-making tool in contraception (SHARECONTRACEPT) concerning: a) Improvement in counselling on hormonal contraception at the medical consultation, measured in terms of decreasing decisional conflict and improving knowledge of available contraceptive options; b) Improvement in adherence to treatment measured in terms of: persistence in the chosen treatment, compliance with dose or procedure of use, and ability to deal with incidents related to the use of the contraceptive method; and decreasing unwanted pregnancies and voluntary interruption of pregnancy.

The SHARECONTRACEPT tool, developed by previous phases of this project, is available at: http://decisionscompartides.gencat.cat/en/decidir-sobre/anticoncepcio_hormonal/

**Methods/design:**

A longitudinal, prospective-type, randomized, controlled community clinical trial, carried out in the clinical contraceptive counselling units of 6 autonomous regions in Spain, with an experimental group and a control group. Description of the intervention: The health professionals participating will be randomly assigned to one of the two groups. Clinicians assigned to the experimental group will perform contraceptive counselling assisted by SHARECONTRACEPT, and those of the control group will follow the conventional contraceptive counselling provided in their clinical unit.

It is planned to study 1708 users (control group *n* = 854 and intervention group *n* = 854), recruited from women who attend the consultations of the health professionals. The selected users will be followed up for one year. The data will be collected through ad-hoc questionnaires, and validated instruments for measuring decisional conflict and adherence to treatment.

**Discussion:**

The results of this study protocol will offer evidence of the effectiveness of a shared decision-making tool, SHARECONTRACEPT, which may prove a useful tool for users and professionals to promote adherence to contraceptive methods.

**Trial registration:**

Clinical Register number ISRCTN5827994. Date: 15/04/2019 (Retrospectively registered)

## Background

In Spain, nine out of ten women of childbearing age, between 15 and 49 years, report that they have had sex, at some time, and two out of ten report having had sex without using any method of contraception. In other words, some two million women would currently be exposed to an unwanted pregnancy in our country (SEC) [1]. In this regard, in 2015 a report was published that stated that approximately 35% of pregnancies in Spain were unwanted (some 190,000 unwanted pregnancies/year) [2].

Half of these unwanted pregnancies in Spain end in voluntary termination (VTP), 93,131 in 2016, according to a report on these terminations by the Spanish Ministry of Health. The report highlights a decline in VTPs in recent years and although it would be nice to think that this is due to better use of contraceptives, the fact that only 10.4% of total VTPs were due to medical reasons is worrying [3]. Also, it should not be forgotten that the role being played by emergency (post-coital) contraception to reduce unwanted pregnancies and the subsequent reduction in the rate of VTPs is not known; according to the latest survey of 2018, presented by the SEC, 30% of respondents reported resorting to emergency contraception, at some time [1].

The condom is the most popular form of contraceptive in Spain: it is used by 29.6% of the population, compared with 17.3% of women who use combined hormonal contraceptives (CHC), the most commonly used hormonal method of contraception across all age brackets. The population continues to rely, therefore, on the condom, a good way to avoid possible contagion by sexually transmitted diseases (STDs), but one that is not a very effective contraceptive in practice when compared with other hormonal methods due to its inconsistent and/or incorrect use [1]. Each year, eighteen out of every 100 women condom users get pregnant during their first year of use compared to nine out of 100 women users of CHC per year, according to Pearl indices corresponding to each method, as published recently by the World Health Organization (WHO) [4].

In the last 30 years, new hormonal contraceptives have been added to the range of existing contraceptive devices, drugs or technologies. Most of these additions have involved modifications or a variety of alternatives to oral hormonal contraception, the best-known and most widely used, but also to other methods such as the progestogen pill (used by 0.1%), combined injectable contraceptives (used by 0.2%), patches (used by 0.7%), and the combined hormonal vaginal ring (used by 2.9%). Long-acting reversible contraception (LARC) methods, including intrauterine devices (IUDs) and the subdermal implant, are the most effective as regards avoiding unwanted pregnancies (Pearl index for implants, the Levonorgestrel IUD and the copper IUD of 0.05, 0.2 and 0.8–2 pregnancies per 100 women/year, respectively) [5]. Based on the latest results of the SEC, in 2018, an increase can be seen in the last year in the use of LARC (9.6% of total users), with respect to the 2016 survey (7.7% of total users) [1, 6].

According to the results of the latest survey on the use of contraceptives in Spain, 1% used an implant, 4.3% had a hormonal IUD and 4.3% had a copper IUD [1]. These methods, in addition to their excellent cost-effectiveness ratio and comfort and safety for the health of women, are highly efficient as they do not influence non-compliance with or the misuse of the method by the user. This aspect differentiates them from other hormonal methods, which, while highly effective in theory, their effectiveness is reduced by the lack of adherence to treatment [1, 4, 7].

The term adherence includes two concepts: compliance with doses (taken correctly in accordance with the drug posology) and persistence in the duration of the prescribed treatment (constant use of treatment over time) [8]. The lack of adherence to contraceptive treatments generally compromises their effectiveness and results in many of the unwanted pregnancies and VTPs, with an estimated annual cost of approximately 80 million euros to the national health system [2].

In a recent review, it was found that in Spain, non-compliant use of contraceptives among users of CHC is common, between 65 and 70% of users forget or delay a pill more than once a month, but moreover, 18% did not use any additional method subsequently and 43% were distressed by the possibility of getting pregnant that month, showing a lack of skills and knowledge as to how to act in the event of an oversight or non-compliance [8]. In addition, there is a high dropout rate from CHC within 12 months of the start of treatment, with figures close to 50%, which are due to the onset of side effects and/or the lack of adherence to treatment [9].

The low use of LARC is not influenced by the lack of adherence, but could be explained by problems related to: a lack of knowledge of such methods, accessibility to them, and the bleeding pattern they present that is not accepted by all women, as they are not informed of it [1, 4, 5, 10]. In this regard, recent SEC survey data show that 45.6% of women acknowledged that their doctor or midwife did not offer them the contraceptive option of a LARC during their counselling consultation, despite the fact that in several of the autonomous communities participating in the population survey, this type of long-acting contraception is financed wholly or in part [1, 10].

The data set out above show the need to encourage the population of potential users of contraceptive methods to get access to balanced and objective information on the different contraceptive methods based on the latest scientific evidence to help them choose the most suitable to their health status and their personal situation [4]. Dehlendorf, the author of an interesting review of how contraceptive advice should be given, stresses the need for clinicians, before such demand, to establish a close and trusting relationship with users.

In recent years, the patient has acquired a more active and responsible role in relation to the management of their health and in the clinical practice. On a daily basis, patients and healthcare professionals face situations in which decisions related to the diagnostic process and/or different treatment options should be taken jointly [11, 12]. To facilitate this, shared decision-making tools (SDMT) have been developed, that promote discussion between patients and healthcare professionals concerning the various treatment options [11–13].

The purpose of this study protocol is to evaluate the effectiveness of an SDMT in open access online digital hormonal contraception during contraceptive counselling consultation, according to the needs of the population.

## Methods/design

### Study framework

The SDMT in hormonal contraception, SHARECONTRACEPT, was previously devised by the authors De Molina, Rubio and De la Flor.

In August 2018, it was published on the “Decisions Compartides” website of the Department of Health of the Generalitat (Government) of Catalonia, in Spanish, Catalan and English: http://decisionscompartides.gencat.cat/en/decidir-sobre/anticoncepcio_hormonal/index.html).

Below, we set out how it was produced, as we consider it relevant as a reference framework for the study:

For the design of the SHARECONTRACEPT SDMT, the criteria of the *International Patient Decision Aid Standards* (IPDAS), an international group of researchers and health professionals set up in 2003 to determine the standard criteria for the development of SDMT in view of their increasing occurrence, were followed [13, 14]. These criteria, grouped in a checklist of 30 items, establish the three blocks for inclusion in SDMTs: clinical content, development process and evaluation of effectiveness [13, 15].

**Fig. 1:**
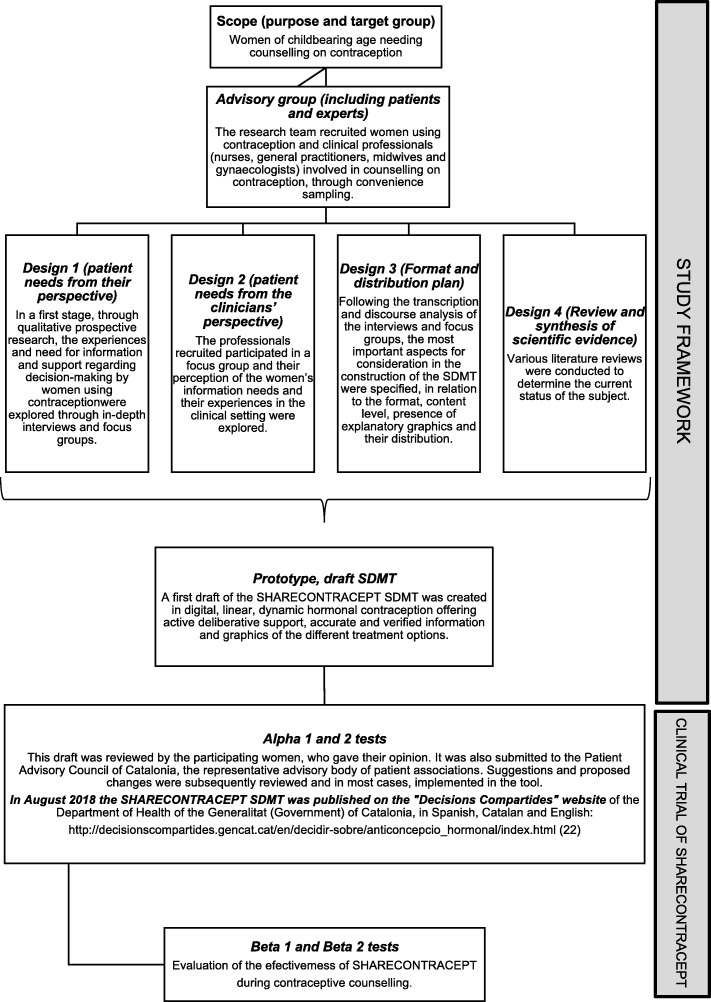
Study framework of the development of the Shared Decision-Making Tool following the stages described by the National Health System (NHS) Agency for Evaluation, Technology and Performance

For the SHARECONTRACEPT development process, the stages described by the National Health System (NHS) Agency for Evaluation, Technology and Performance were followed for SDMT evaluation and validation [15]. Then, the development process is described in stages (Study framework flowchart of the design of SHARECONTRACEPT, Fig. 1).

### Clinical trial of SHARECONTRACEPT

#### Hypothesis

Advice on hormonal contraception at the physician’s office, with the support of the SHARECONTRACEPT SDMT, encourages dialogue and the exchange of information between the woman and the clinician, which can help select a contraceptive method more in accordance with her preferences, needs, priorities and characteristics, thus improving adherence to the method of choice.

#### Aims and objectives

The purpose of this project is to evaluate the effectiveness of the SHARECONTRACEPT SDMT during contraceptive counselling in six autonomous communities of Spain

1. To determine whether the use of SHARECONTRACEPT *improves* adherence to the chosen contraceptive treatment, in relation to compliance with dose or method of use and persistence in the duration of the prescribed treatment,
2. To assess whether SHARECONTRACEPT increases the ability to cope with incidents related to the use of the method,
3. To assess the social impact of using SHARECONTRACEPT in relation to decreasing unwanted pregnancies and voluntary termination of pregnancy (VTP),
4. To analyse whether SHARECONTRACEPT decreases the woman’s conflict in view of the choice of contraceptive method,
5. To assess whether the use of SHARECONTRACEPT helps clinicians to better understand the various hormonal contraceptive options available and their characteristics (risks, benefits and consequences), and.
6. To analyse whether the use of SHARECONTRACEPT changes the pattern of use of hormonal contraception in the population towards more effective methods.

#### Design

The research is approached as a longitudinal, prospective community clinical trial. While in the conventional format of clinical trials allocation is performed on the participating subjects themselves, in the community format, clusters are assigned to which the units of analysis belong.

**Fig. 2:**
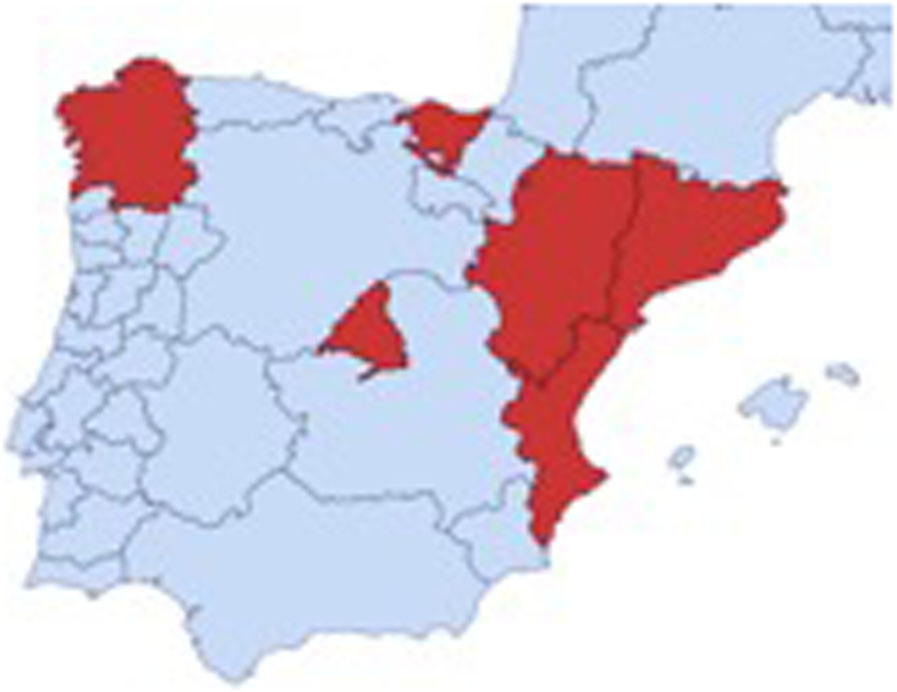
Map of participating communities where the clusters were selected. Created by the author

In this research, the clusters are the professionals of the Clinical Units of the six participating Spanish autonomous communities: Madrid, Aragon, Galicia, the Basque Country, Valencia and Catalonia (see Fig. 2). The units of analysis are the women attending the consultation of the professionals between 1 July 2019 and 30 June 2020.

### Clusters sample

Firstly, the clusters were selected. The researchers responsible for each of the six autonomous communities in Spain recruited them by means of convenience sampling. During the first quarter of 2019, professional clinicians giving contraceptive counselling (gynaecologists, midwives, family doctors and/or nurses) from the different Spanish autonomous communities participating were invited to take part. One hundred and sixty-four (164) professionals agreed to participate and they were randomly assigned using the EPIDAT program (single sampling), to one of two groups:

1. Experimental Group (EG), professionals who will perform counselling with the support of the SHARECONTRACEPT SDMT, and
2. Control group (CG), professionals who will perform clinical counselling as they had until then (conventional contraceptive counselling).

Starting from this context, it was decided to create the Web page: Sharecontracept.com, through which the professionals can easily access the three different environments in which they are to participate.

#### 1-virtual campus

In order, insofar as possible, to standardize the procedure to be followed given that the different professional profiles and broad geographical spread, the Foundation of the Rovira i Virgili University in Tarragona (Spain) designed a 60-h online course for the professionals involved in the project.

This course is to be implemented prior to starting the fieldwork. There are two courses, one for the CG and the other for EG. The professionals of each group will receive an email with a link in order to access the corresponding SHARECONTRACEPT virtual course with a username and a password. Once inside the course, and after a brief presentation, they will be asked to perform a test of knowledge on contraception at the beginning and at the end of the course, to assess whether SHARECONTRACEPT improves the knowledge of the professionals who use it. After the first test, the system will allow them access to the rest of the course environment, where, among others, they will find forums through which they can contact the research team.

In addition, through documents and videos, they will get access to the contents of the different subjects (see Table 1), depending on whether they are professionals assigned to EG (they will be able to view the six subjects from the beginning) or CG (during the fieldwork they will only be able to view subjects 2 and 6, although they will be able to get access to the rest once sample collection has finished).

**Table 1:** Moodle course table of contents

Theme 1	The shared decisions model
Theme 2	The SHARECONTRACEPT project
Theme 3	Which are the WHO’s eligibility criteria
Theme 4	Keys to giving good counselling
Theme 5	Videos with examples of how to counsel with the support of SHARECONTRACEPT
Theme 6	Instructions on how to implement the information collection platform of the SHARECONTRACEPT Project

#### 2- user registration

Having reviewed the course contents, the professional will be requested to register with the data collection platform.

#### 3-platform

Finally, via this icon, access to the data collection base is granted, using a username and personal password, to start inputting cases (units of analysis).

### Unit analysis sample

#### Sample size

In a second stage the units of analysis are recruited. A sample of 1708 women is established (Table 2). The calculation was performed using the EPIDAT program to test hypotheses in a model of comparison of proportions in independent groups. A possible loss of 25% of the sample is estimated, and so by applying the correction factor where R is the expected proportion of losses, the following sample size is finally envisaged:

**Table 2:** Study sample size calculated using the EPIDAT program

Power (%)	Sample size^*^ (+ correction factor for expected losses)		
	EG	CG	Total
80.0	854	854	1708

Sample sizes to apply the χ2 test with Yates continuity correction

*An α risk of 0.05 and a β risk of 0.2 (80% power) are assumed, standard values in such studies

##### Sample selection criteria

The sample will be selected from the women attending the practice of one of the professionals from either CG or EG requesting counselling on contraception or when such need is found during the stipulated period (1 July 2019 to 30 June 2020) and who fulfil the following **inclusion criteria**: have completed their primary education and be competent in Spanish and/or Catalan (the languages of SHARECONTRACEPT, although it is also in English); be between 16 and 49 years of age, have internet access, and agree to participate voluntarily in the study. **Women are excluded** who wish to have children within one year of the date of consultation or have a history of: stroke, acute myocardial infarction, thrombosis, breast cancer (< 5 years), liver cirrhosis or liver tumour.

##### Information collection questionnaires

Information on each of the women will be collected at four times during a year, from two online questionnaires developed ad hoc: an in-person survey and a telephone survey (to be conducted 3 times: at one month, at 6 months, and one year following the in-person consultation).

The main variable is adherence to treatment (compliance with the dose and method of administration and persistence in the duration of the prescribed treatment) and the secondary variables include *the woman’s sociodemographic characteristics, her medical, obstetrical and gynaecological history, her contraceptive method, decisional conflict, and variables of the professional* (see Table 3).

**Table 3:** Variables collected in the study

Main variable	*Adherence to treatment:*
	• Compliance with dose and method of administration and
	• Persistence in the duration of the prescribed treatment
Secondary variables	*Woman’s sociodemographic characteristics:*
	• Age
	• Level of education
	• Occupation
	• Marital status/partner
	*Contact details*
	*• Telephone*
	*• Telephone 2*
	*Medical history:*
	*•* Smoker
	*•* HBP
	*•* Uterine malformations
	*Obstetric and gynaecological history:*
	*•* Menstrual pattern
	*•* Miscarriage
	*•* VTP
	*•* Living children
	*Contraceptive method*
	*•* Contraceptive method considered by the patient before counselling
	*•* Experiences with other methods
	*•* Woman’s attitude towards compliance (Morisky-Green treatment adherence test)
	*•* Contraceptive finally chosen
	*•* Incidents with the chosen method
	*•* Incident management
	Decisional conflict
	The woman’s decisional conflict in view of the choice of contraceptive method (O’Connor Scale) https://decisionaid.ohri.ca/eval_dcs.html
	And of the professional:
	*•* Satisfaction with the counsellor or clinician with the use of the digital SDMT (Likert scale)
	*•* Test of knowledge prior to and after fieldwork

**The statistical approach** will consist of a set of bivariate and multivariate descriptive analyses and the application of logistic regression models to determine the extent to which the likelihood of changing birth control method and the likelihood of choosing one particular method are linked to certain specified variables. Losses are considered as women that do not complete the follow-up of the protocol to which they were assigned or that do not to remain until the end of the analysis.

### Study limitations

The fact that the two groups (EG and CG) may come into contact at the same health centre or unit could result in professionals who must continue to provide counselling in a conventional manner (CG) potentially modifying how they behave if they find out about or get access to SHARECONTRACEPT due to comments by their EG peers. To avoid this insofar as possible, during training EG will be asked to keep absolute confidentiality.

Different autonomous communities, different sexual and reproductive healthcare policies, different funding schemes for contraceptive methods, different professionals giving counselling with different levels of training (midwives, nurses, gynaecologists and family physicians) could be limitations, and so these variables must be duly taken into account when analysing the results.

## Discussion

It is recommended, during counselling on contraception, to use a shared decision-making approach focusing on ascertaining the user’s values and preferences, which helps facilitate qualified information on each contraceptive method and according to their needs in order to promote adherence to the chosen contraception and advise in the event of the occurrence of side effects, among others [16].

Before choosing a particular method there are aspects related to the potential user and the characteristics of each of the methods that must be clearly defined. To begin with, there is a need for a good anamnesis including the woman’s age, previous knowledge of the subject, information about her sexual partner, the purpose of contraception, personal preferences and predisposing factors for poor compliance in accordance with the method [17]. Regarding the method, the woman must be familiar with the main features of the existing methods; their effectiveness, side effects and safety, reversibility, possible non-contraceptive benefits, ease or complexity of use, impact on intercourse, and price, among others [18].

Dehlendorf warns that many women express dissatisfaction with the counselling they receive from clinicians [16], and other studies have found that clinicians show inaccurate knowledge of contraceptive methods and/or possess outdated information [16, 19, 20]; it would also appear that the decision made by women is strongly influenced by the mention or recommendation of specific methods by the clinicians providing counselling, in a paternalistic healthcare environment [11, 16]. SDMTs have been created to change this. Specifically, SHARECONTRACEPT has been devised to help clinicians and users in the processes of learning, counselling and choice of the contraceptive method that best suits each person and situation.

This project enables testing by users and clinicians in the daily practice and within the health system whether this SDMT in hormonal contraception is effective, giving them all an opportunity to review it and comment on it, which are key aspects of the development process of an SDMT [15].

## Data Availability

The study is ongoing. Data will be available when the study ends (31/12/2021).

## Abbreviations

CG: Control group
CHC: Combined hormonal contraception
EG: Experimental group
IPDAS: International Patient Decision Aid Standards
IUD: Intrauterine devices
LARC: Long-Acting Reversible Contraception
SDMT: Shared Decision-Making Tools
SEC: Sociedad Española de Contracepción (Spanish Society of Contraception)
STD: Sexually transmitted diseases
VTP: Voluntary termination of pregnancy
WHO: World Health Organization
